# The pyrethroid insecticide deltamethrin disrupts neuropeptide and monoamine signaling pathways in the gastrointestinal tract

**DOI:** 10.1093/toxsci/kfaf076

**Published:** 2025-06-26

**Authors:** Alexandria C White, Ian N Krout, Sabra Mouhi, Lisa Blackmer-Raynolds, Jianjun Chang, Sean D Kelly, William Michael Caudle, Timothy R Sampson

**Affiliations:** Department of Cell Biology, Emory University School of Medicine, Atlanta, GA 30322, United States; Aligning Science Across Parkinson’s (ASAP) Collaborative Research Network, Chevy Chase, MD 20815, United States; Department of Cell Biology, Emory University School of Medicine, Atlanta, GA 30322, United States; Aligning Science Across Parkinson’s (ASAP) Collaborative Research Network, Chevy Chase, MD 20815, United States; Department of Cell Biology, Emory University School of Medicine, Atlanta, GA 30322, United States; Department of Cell Biology, Emory University School of Medicine, Atlanta, GA 30322, United States; Aligning Science Across Parkinson’s (ASAP) Collaborative Research Network, Chevy Chase, MD 20815, United States; Department of Cell Biology, Emory University School of Medicine, Atlanta, GA 30322, United States; Department of Cell Biology, Emory University School of Medicine, Atlanta, GA 30322, United States; Gangarosa Department of Environmental Health, Rollins School of Public Health, Emory University, Atlanta, GA 30322, United States; Department of Cell Biology, Emory University School of Medicine, Atlanta, GA 30322, United States; Aligning Science Across Parkinson’s (ASAP) Collaborative Research Network, Chevy Chase, MD 20815, United States

**Keywords:** pyrethroids, gastrointestinal tract, enteroendocrine cells, serotonin, neuropeptides

## Abstract

Enteroendocrine cells (EECs) are a rare cell type of the intestinal epithelium. Various subtypes of EECs produce distinct repertoires of monoamines and neuropeptides, which modulate intestinal motility and other physiologies. EECs also possess neuron-like properties, suggesting a potential vulnerability to ingested environmental neurotoxicants. One such group of toxicants is pyrethroids, a class of prevalent insecticides used residentially and agriculturally. Pyrethroids agonize voltage-gated sodium channels (VGSCs), inducing neuronal excitotoxicity, and affect the function of monoamine-producing neurons. Given their anatomical location at the interface with the environment and their expression of VGSCs, EECs likely represent a vulnerable cell type to oral pyrethroid exposure. In this study, we used the EEC cell line, STC-1 cells, to evaluate the effects of the common pyrethroid deltamethrin on the functional status of EECs. We find that deltamethrin impacts both the expression of serotonergic pathways and inhibits the adrenergic-evoked release of an EEC hormone, glucagon-like peptide 1, in vitro. In a mouse model of oral exposure, we found that deltamethrin induced an acute, yet transient, loss of intestinal motility in both fed and fasted conditions. This constipation phenotype was accompanied by a significant decrease in peripheral serotonin production and an inhibition of nutrient-evoked intestinal hormone release. Together, these data demonstrate that deltamethrin alters monoaminergic signaling pathways in EECs and regulates intestinal motility. This work demonstrates a mechanistic link between pyrethroid exposure and intestinal impacts relevant to pyrethroid-associated diseases, including inflammatory bowel disease, neurodegenerative disease, and metabolic disorders.

Enteroendocrine cells (EECs) are sensory transducing cells of the gastrointestinal (GI) epithelium. Physically located at the interface of the intestinal-luminal environment, EECs are readily exposed to numerous factors, including intrinsic microbiome components and exogenous environmental and dietary molecules. In response to such stimuli, subsets of EECs produce various monoamines and neuropeptides, including serotonin (5-hydroxytryptamine [5-HT]), cholecystokinin, peptide YY, and glucagon-like peptide 1 (GLP-1). These signaling molecules act to modulate intestinal motility, regulate blood glucose, and transmit satiety signals to the brain (Brierley et al. 2021; Hayashi et al. 2023). Although the role of EECs in microbial and nutrient sensing is well described (Chimerel et al. 2014; Zelkas et al. 2015; Larraufie et al. 2018; Martin et al. 2019; Ye et al. 2021), whether and how EECs respond to common environmental toxicants is largely unknown.

Pyrethroids are a common insecticide used residentially (e.g. home pest control, gardening, and lice treatment), in veterinary medicine (e.g. flea and tick treatment), and in agricultural industries. Their prevalent and large-scale use in food production and agriculture results in widespread pyrethroid detection within foodstuffs and water, ultimately leading to chronic, low-dose oral exposures to the human population (ATSDR 2003). In rare cases of acute toxicity, high-level exposure leads to neurological defects, including seizures, discoordination and imbalance, and cognitive impairment (He et al. 1989). These central nervous system (CNS) impacts are likely due to pyrethroid actions on their main molecular targets, voltage-gated sodium channels (VGSCs). Pyrethroids are VGSC agonists, leading to their prolonged hyperactivation (Soderlund et al. 2002). Although Type I pyrethroids act primarily on VGSCs, Type II pyrethroids simultaneously antagonize GABA_A_ receptors, furthering neuronal excitation by preventing chloride-mediated inhibition. Through these mechanisms, experimental models have demonstrated that pyrethroid exposure leads to significant disruption of monoamine signaling in neurons, including serotonin and dopamine (Kirby et al. 1999; Elwan et al. 2006; Hossain et al. 2013; Richardson et al. 2015; Rodríguez et al. 2016). However, the actions of pyrethroids on non-neuronal cells of the periphery are less described.

Given their location within the intestinal epithelium, EECs represent a likely first cellular target of pyrethroids and other ingested toxicants. Their potential vulnerability to pyrethroid toxicity is highlighted by their expression of pyrethroid-sensitive VGSCs. Using deltamethrin, a prevalent Type II pyrethroid insecticide, we assessed impacts to monoamine pathways and hormonal release in both an EEC culture model and in a murine oral exposure model. We find that deltamethrin dose-dependently interferes with the expression of monoamine pathways in EECs in vitro and induces acute, but transient, intestinal dysmotility in vivo. Correspondingly, we observe that intestinal serotonin concentrations are significantly and transiently reduced after acute deltamethrin exposure. Lastly, we demonstrate that stimulated release of EEC neuropeptides is suppressed by acute deltamethrin exposure both in vitro and in vivo and is chronically associated with increased food intake. Overall, these data demonstrate a significant impact of a prevalent pyrethroid on EEC status and GI function, suggesting a mechanistic link for pyrethroid-associated diseases that impact the GI tract, including inflammatory bowel disease (IBD) (Ma et al. 2023), metabolic syndrome (Wang et al. 2011; Hansen et al. 2014; Bao et al. 2020; Wei et al. 2023), and Parkinson’s disease (PD) (Gillette and Bloomquist 2003; Kannarkat et al. 2015; Xiong et al. 2016).

## Materials and methods

### Chemicals and treatments

For cell culture experiments, deltamethrin (Cat#: N-11579-250MG, purity 99%, Chem Service, Inc, Westchester, Pennsylvania) and bioallethrin (Cat#: P-664N, purity 98.7%, AccuStandard, Inc, New Haven, Connecticut) were dissolved in sterile dimethyl sulfoxide (DMSO, cell culture grade) to form a stock concentration of 100 mM that was aliquoted and stored at −20°C until use. For mouse experiments, deltamethrin was dissolved in filter-sterilized corn oil (Mazola) and prepared fresh 1 day prior to each use for acute experiments or every 3 uses for chronic experiments, wherein it was stored in the dark at room temperature (RT). Laboratory-grade acetone was used to facilitate deltamethrin’s dissolution and was allowed to evaporate overnight. For cell culture, glucose and epinephrine were both purchased from Sigma (Glucose Cat#: G8270-1KG, purity 99.5%; Epinephrine Cat#: E4250-1G, purity 99%), dissolved in PBS, and aliquoted for storage in −20°C. Calcium-free media (Gibco Cat#: 21068028) was supplemented with 0.1 mM EDTA, filter-sterilized, and stored at 4°C. Ensure^®^ (Abbott Pharmaceuticals) mixed-meal nutrient mixtures were prepared fresh on the day of use and mixed with 6% carmine red dye with 0.5% methylcellulose (Sigma Cat#: C1022 and M7027, respectively) prior to gavage. Unless stated otherwise, all stock solutions were filter-sterilized prior to use and were of pharmaceutical or food grade.

### Animal husbandry

Ten- to twelve-week old C57BL/6J male and female mice (The Jackson Laboratory, RRID: IMSR_JAX: 000664), as indicated, were co-housed according to treatment group in Emory University’s Whitehead Biomedical Research Building rodent facility. Mice were provided standard food and water ad lib, except when noted for mixed-meal nutrient stimulation experiments, under a 12 h light/dark cycle with lighting at 7 AM. At the experimental endpoints, 4 to 24 h or 12 weeks following final treatments or exposures, mice were humanely euthanized via cardiac perfusion with sterile PBS after deep isoflurane anesthesia. All animal husbandry and experimental procedures were performed in accordance with Emory's Institutional Animal Care and Use Committee (IACUC) protocol #201900030.

### Oral exposures

For acute exposures, mice were orally gavaged once with 50 µl of 3 mg/kg deltamethrin (Chem Service, Cat#: N-11579-250MG) dissolved in filter-sterilized corn oil or 50 µl of filter-sterilized corn oil alone as a vehicle control using a sterilized metal feeding needle (22G) as described previously (White 2025c). This dose aligns with prior work (Elwan et al. 2006; Vester et al. 2019; Hossain et al. 2022a, 2022b) and falls within previously reported no observable effect and no observable adverse effect limits, defined by the US EPA (EPA 2018). Mice were monitored closely for overt signs of acute toxicity, none of which were observed throughout our studies. Corn oil was selected as a vehicle for its longstanding use in the field of pyrethroid toxicology (Ito et al. 1993; Crofton et al. 1995; Anadon et al. 2006; Pine et al. 2008; Weiner et al. 2009; Hossain et al. 2022a) and relevance to modeling oral exposures given its use as food (Dupont et al. 1990). For chronic exposure experiments, mice were gavaged once weekly for 12 weeks. Subsequent intestinal assays were conducted within consistent timeframes to account for circadian rhythm. For acute experiments, gavages occurred every 7 min beginning at 10 AM for 4 h exposures or 3 PM for 24 h exposures, whereas carmine/mixed-meal nutrient gavages (100 µl) occurred every 7 min starting at 12:30 PM, and fecal output and any motor behaviors occurred in the afternoons at 2 PM. For chronic experiments, gavages occurred at 1 PM once per week, whereas fecal output occurred the following morning at 10 AM, and tests of total GI transit occurred at 0-, 6-, and 12-weeks post-exposure and lasted from 10 AM until 6 PM. Food intake was measured only under chronic treatment conditions each week on the day prior to deltamethrin or vehicle gavage by weighing the amount of food consumed each week per cage, where each cage contained 4 mice. Therefore, food intake per cage = amount of food added the prior week−amount of food remaining at the end of the week.

### Intestinal behaviors

#### Fecal output

Performed as previously described (Hamilton et al. 2024b). At the indicated timepoints (Acute: 4 h or 24 h after single oral gavage; Chronic: 24 h after each oral gavage), mice were placed in a sterile 1 L plastic container to count the number of fecal pellets produced every 5 min for 30 min.

#### Intestinal motility assay

Performed as described previously (White 2024a). At the indicated timepoints, mice were orally gavaged with 100 µl 6% carmine red dye 1.5 h prior to sacrifice to visually track motility. During tissue collection, the distance traveled (cm) by the carmine dye was recorded as a percentage of the total length of the GI tract (small intestine length + large intestine length, excluding cecum). If the dye was in the cecum, it was recorded as traveling 100% of the small intestine length. In a second set of experiments, Ensure (Abbott Pharmaceuticals) was pre-mixed with 6% carmine red dye prior to each mouse receiving 100 µl of the mixture 1.5 h prior to sacrifice.

#### Total intestinal transit assay

Performed as previously described in detail (Hamilton et al. 2024a). After 1 h of habituation in an isolated behavioral testing room, mice were orally gavaged with 100 µl carmine red dye solution (6% carmine red dye mixed with 0.5% methylcellulose). One hour later, mice were split into separate cages where they were housed individually with access to food and water but no bedding. Mice were observed every 15 min for up to 8 h or until a red fecal pellet was produced, at which point mice were returned to their home cage. The difference in time at which the mouse is gavaged and a red pellet is produced equals total intestinal transit time and was recorded in hours.

### Cell culture

The mouse (*Mus musculus*) secretin tumor cell line (STC-1, RRID: CVCL_J405) at passage 29 was purchased from the American Type Culture Collection (ATCC, Cat#: CRL-3254) and cultured as described in detail (Hurley and White 2024). Cells were seeded in 24- or 96-well plates at a density of 80,000 or 20,000 cells/well, respectively. Cells were cultured in DMEM/F12 media with GlutaMAX (GIBCO, Cat#: 10565018) supplemented with 10% heat-inactivated fetal bovine serum (Gibco, Cat#: 16140071) and 2% penicillin/streptomycin (5,000 U/ml, Gibco, Cat#: 15070063), modified from work described previously (McCarthy et al. 2015). Cells were incubated in a 5% CO_2_ humidified chamber held at 37°C, and passage numbers were maintained between 30 and 35. Cells were passaged via 5-min incubation in 5 ml 0.25% Trypsin-EDTA (Gibco, Cat#: 25200072). Media was changed every other day until treatments, which occurred 3 to 4 days after seeding, once cells reached ∼80% confluency.

### Cytotoxicity and cell viability assays

#### Lactate dehydrogenase assay

Chemical-mediated cell death was assessed using the colorimetric CytoTox 96 Non-Radioactive Cytotoxicity Assay (Promega, Cat#: G1780), which measures lactate dehydrogenase (LDH) released into the supernatant, according to the manufacturer’s instructions and described in detail previously (White 2024b). LDH was quantified 24 h after treatments. Cells were grown to confluency (approximately 48 to 72 h after seeding at 10 k cells/well in a 96-well plate) and maintained at 37°C until treatments. Maximum LDH release was measured by adding 10 µl lysis buffer per 100 µl media to a subset of untreated control wells and allowing them to incubate for 45 min. Afterward, 50 µl of supernatant from each well was collected and placed in a new 96-well plate for LDH quantification along with 50 µl of CytoTox 96 Reagent and incubated for 30 min at RT in the dark. Stop solution was added immediately prior to absorbance reading, which was measured at 490 nm using a spectrophotometer.

#### 3-(4,5-dimethylthiazol-2-yl)-2,5-diphenyltetrazolium bromide assay

Cell viability was assessed as described previously (White 2025b) using the CyQUANT MTT Cell Viability Assay (Invitrogen, Cat#: V13154), which measures cellular redox potential through their ability to reduce the water-soluble MTT (3-(4,5-dimethylthiazol-2-yl)-2,5-diphenyltetrazolium bromide) into an insoluble formazan product. Cells were treated for 24 h prior to assessment of cell viability. Cells were resuspended in 100 µl of fresh culture medium (phenol-free), and 10 µl of the 12 mM MTT stock solution was added to each well, including negative-control wells without cells. Cells were then incubated at 37°C for 3 h. All but 25 µl of culture media was removed prior to the addition of 50 µl DMSO with repeated pipetting to ensure the solubilization of the formazan products. After another 37°C incubation for 10 min, the absorbance was read at 540 nm using a spectrophotometer.

### Gene expression analysis

#### RNA extraction

At confluency, STC-1 cells were treated with 0.01% DMSO vehicle control or 12.5, 25, 50, or 100 µM deltamethrin. Approximately 24 h later, cells were collected for RNA extraction as described in detail previously (Tansey and White 2024b). Briefly, cells were washed gently once with PBS, and 250 µl Trizol (Zymo Research, Cat#: R2050-1-200) was added to each well for RNA extraction using the Qiagen RNeasy Mini Kit (Cat#: 74106). In brief, cell lysates were homogenized by sonication (QSonica, ∼35 to 45 setting) for 3 to 5 s before one-fifth volume of chloroform was added to each sample and shaken for 15 s. After sitting for 2 min at RT, samples were centrifuged at 12,000 rpm for 15 min at 4°C. The upper aqueous clear layer was transferred to a QIAshredder (Qiagen, Cat#: 79656) and centrifuged for 2 min at full speed at RT. The flow-through was combined with a half volume of 70% ethanol and transferred to an RNeasy spin column. Samples were centrifuged briefly for 30 s at 10,000 rpm, and the flow-through was discarded; 700 µl Buffer RW1 was added to the spin column prior to centrifuging again for 30 s at 10,000 rpm. Flow-through was discarded, and 500 µl buffer RPE was added to the spin column, followed by centrifuging for 30 s at 10,000 rpm. This step was repeated, but instead centrifuged for 2 min before discarding the flow-through. The RNeasy spin column was then placed in a new collection tube and centrifuged for 1 min at full speed to collect any excess liquid. Finally, the spin column was placed in a new 1.5 ml Eppendorf tube, 30 µl RNase-free water was added, and samples were centrifuged at 10,000 rpm for 1 min to elute RNA. RNA concentrations for each sample were measured using a NanoDrop.

#### cDNA conversion

RNA was converted to cDNA using the iScript cDNA Synthesis Kit (Bio-Rad, Cat#: 1708891) following the manufacturer’s instructions and described in detail previously (Dusseldorf and White 2024). Briefly, samples were diluted to 50 ng/µl up to a total of 1 µg RNA. To achieve a 20 µl reaction volume, 4 µl of 5× iScript Reaction Mix and 1 µl iScript Reverse Transcriptase was added for every 15 µl of sample. The complete reaction mix was then incubated in a thermal cycler as follows: Priming for 5 min at 25°C, reverse transcription for 20 min at 46°C, inactivation for 1 min at 95°C, and an optional hold step at 4°C.

#### Real-time quantitative PCR

Real-time quantitative PCR was performed for gene expression analysis according to a previously described protocol (Tansey and White 2024a). cDNA was diluted 1:10 to reach a final amount of 10 ng cDNA per reaction. Primer pairs were diluted from 100 µM stocks to 10 µM combined working stocks. Per sample and gene of interest, a 12 µl reaction mix was created: 6 µl SYBR Green PCR Master Mix (Thermo Fisher Scientific, Cat#: 4309155), 2 µl diluted primer pairs, 2 µl DI H_2_O, and 2 µl diluted cDNA. Each sample was tested in technical duplicate, and appropriate controls and blanks were used (no-template control, cDNA only, DI H_2_O only). All primer pair sequences are reported in Table S1 and the available key resource table in the Zenodo repository under accession 14803680 (doi.org/10.5281/zenodo.14803680).

### Bulk RNA sequencing

Full service, bulk RNA sequencing was performed through Novogene Corporation, Inc. (Sacramento, CA). Detailed RNA preparation, sequencing, analysis methods, and source data are found in the Zenodo repository under accession numbers 14775919 and 14803680 (doi.org/10.5281/zenodo.14775919 and doi.org/10.5281/zenodo.14803680).

#### Library preparation

A total input of 1 µg RNA was used to generate sequencing libraries using NEBNext UltraTM RNA Library Prep Kit for Illumina (NEB, USA), per manufacturer’s instructions. Following mRNA enrichment using poly-T oligo-attached magnetic beads, RNA was fragmented using divalent cations under elevated temperature in NEBNext First Strand Synthesis Reaction Buffer (5×). cDNA was synthesized first with random hexamer primers and M-MuLV Reverse Transcriptase (RNase H−) and amplified by DNA Polymerase I and RNase H. NEBNext Adaptors were ligated, and ∼150 to 200 bp fragments were purified with the AMPure XP system (Beckman Coulter, Beverly, USA). Fragments were amplified with Phusion High-Fidelity DNA polymerase, Universal PCR primers and Index (X) Primer and amplified products purified (AMPure XP system) assessed for quality on the Agilent Bioanalyzer 2100 system.

#### Clustering and sequencing

CBot Cluster Generation System using PE Cluster Kit cBot-HS (Illumina), according to the manufacturer’s instructions, was used to cluster samples prior to paired-end sequencing on the Illumina platform NovaSeq X Plus Series (PE150) to a depth of 20M reads (6G). Raw sequencing reads are available in the NIH SRA database at accession number PRJNA1218260.

#### Quality control

Raw data in FASTQ format were first processed through *fastp* (RRID: SCR_016962; v0.23.1) to remove adapter and poly-*N* sequences and reads with low quality from raw data, as described in detail along with quality control files in doi.org/10.5281/zenodo.14775919.

#### Genome alignment and analysis

Reference genome (*M. musculus*, GRCm39/mm39) and gene model annotation files were downloaded from genome website browser (NCBI/UCSC/Ensembl) directly. Paired-end clean reads were aligned to the reference genome using Hisat2 (RRID: SCR_015530; v2.0.5). Mapped reads were assembled using StringTie (RRID: SCR_016323; v1.3.3b) (Pertea et al. 2015). featureCounts (RRID: SCR_012919; v1.5.0-p3) (Liao et al. 2014) was used to quantify mapped reads and calculate RPKM for differential analysis. Differential expression analysis was performed using DESeq2 R package (RRID: SCR_015687; v1.20.0) (Love et al. 2014). The resulting *P* values were adjusted using the Benjamini and Hochberg’s approach for controlling the False Discovery Rate. Genes with an adjusted *P* value <0.05 and |log_2_(foldchange)| ≥ 1 found by DESeq2 were assigned as differentially expressed. Pathway enrichment was performed using Metascape (RRID: SCR_016620; v3.5.20240901; https://metascape.org) (Zhou et al. 2019). Enriched terms, defined by Metascape, are selected using Fisher’s exact test with a *P*-value cutoff threshold of 0.02.

All analysis files, including aligned sequences, differential gene expression, and Metascape source inputs and outputs are available in the Zenodo repository under accession numbers 14775919 and 14803680 (doi.org/10.5281/zenodo.14775919 and doi.org/10.5281/zenodo.14803680).

### Protein quantifications

#### ELISAs

ELISAs were performed according to the manufacturer’s instructions and described in detail previously (Koletsi 2023). Active GLP-1 release in STC-1 cell supernatant was detected with a GLP-1 (Active) ELISA kit (Millipore, Cat#: EGLP-35K). Samples were diluted 10-fold into kit-provided Assay Buffer prior to adding 200 μl sample/well. The plate was incubated overnight at 4°C followed by 5 washes prior to the addition of 200 μl Detection Conjugate, which was incubated on a slow shaker for 2 h at RT. The plate was washed 3 times, and Substrate was added to each well for a final 20-min incubation in the dark at RT. Once sufficient fluorochrome was generated, 50 μl stop solution was added before reading the plate at an excitation/emission wavelength of 355 nm/460 nm.

#### Multiplexed ELISAs

To detect gut hormones in serum, blood derived from cardiac puncture was phase separated into serum via Vacuette tubes (Greiner-Bio One, Cat#: 454243P) after centrifugation at RT for 10 min at 1,800 rcf and stored at −80°C until sample analysis. Multiplexed ELISAs were performed on indicated serum samples through the Emory University Multiplexed Immunoassay Core. Experiments were performed in duplicate using the U-PLEX Metabolic Hormones Combo 1 for mouse (Meso Scale Discovery, Rockville, MD, USA, Cat#: K15306K-2) with “blank” replicates that served as negative controls, according to the manufacturer’s instructions (White and Chang 2025). All samples were diluted 2-fold for analyses. Heatmapper (RRID: SCR_016974) was used to visualize gut hormone levels in each treatment condition using the average linkage clustering method and the Euclidean distance measurement method (Babicki et al. 2016).

### High-performance liquid chromatography

High-performance liquid chromatography (HPLC) was performed on indicated samples (ileum, colon, serum) through the Emory University HPLC Bioanalytical Core, and as described previously (White 2025a). Samples were resuspended in 300 µl ice-cold 0.1 M PCA, 0.1 mM EDTA, and sonicated on dry ice using probe sonication. Pulses were 1 s on, 10 s off, and used an amplitude of 25% for 20 to 30 s (QSONIC Q500A, Newtown, CT). The homogenated samples were then centrifuged at 13,000 × *g* for 15 min at 4°C. Sample supernatants were transferred into new 0.22 µM PVDF microcentrifuge filter tubes and filtered through a spin filter at 5000 × rpm for 5 min at 4°C. Reverse-phase HPLC with electrochemical detection was used to measure monoamine concentrations. Protein pellets were dissolved in 500 µl of 2% sodium dodecyl sulfate. Protein quantification was performed in triplicate in 96-well microplates with SpectraMax M5e spectrophotometer (Molecular Devices, Sunnyvale, CA) using the Pierce BCA Protein Assay Kit (Thermo Scientific, Cat #A55864). Monoamines were quantified using the ACQUITY ARC system equipped with a 3465 electrochemical detector (Waters). Separations were performed using an Xbridge BEH C18, 2.5 µm, 3 × 150 mm column (Waters) at 37°C. The mobile phase contained 100 mM citric acid, 100 mM phosphoric acid, pH 3.3, 0.1 mM EDTA, 525 mg/l 1-octanesulfonic acid, and 7% acetonitrile. The detection flow cell was SenCell with a 2 mm GC WE, and the cell potential was set at 800 mV with a salt bridge reference electrode. The AST position was set at 1, whereas the ADF was 0.5 Hz. The needle was washed with water, and the pump piston was washed with 15% isopropanol; 20 µl of each sample was injected before being eluted isocratically at 0.6 ml/min. The analytes were identified by matching criteria from retention time measures to known standards (Sigma Chemical Co., St Louis, MO). Compounds were quantified by comparing peak areas to those of standards.

### Statistical analyses

All datasets were analyzed using GraphPad Prism 10 statistical software (RRID: SCR_002798; v10.4.1). Comparisons between 2 groups were generated using two-tailed *t*-tests for normally distributed data or by Mann–Whitney nonparametric tests for non-normally distributed data. Comparisons between more than 2 groups were analyzed with one-way (1 independent variable) or two-way (2 independent variables) ANOVAs and follow-up post-hoc tests. Dunnett’s multiple comparisons test was used to compare means to a control mean, Šídák’s multiple comparisons test was used to compare pre-selected means to one another, and Tukey’s multiple comparisons test was used to compare all means to one another. Where indicated, multiple *t*-test comparisons were performed. Outliers were removed according to the ROUT outlier identification test.

All source data and statistical outputs are included with this manuscript as Supplemental Material and are also available at the Zenodo repository under accession number 14803680 (doi.org/10.5281/zenodo.14803680). Raw sequencing files are deposited in the NIH SRA, project number PRJNA1218260, and aligned reads and all analysis outputs are in Zenodo under accession 1477919 (doi.org/10.5281/zenodo.14775919).

## Results

### Deltamethrin dysregulates monoamine pathways in EECs

To address the effects of deltamethrin on EECs, we first used the STC-1 cell line, a murine transformed cell line that is broadly representative of EEC subtypes (McCarthy et al. 2015). We observed that this line expresses a set of VGSCs, namely *Scn2a-Scn11a*, excluding *Scn5a* (Fig. S1A). Other than *Scn2a* and *Scn9a*, these VGSCs are sensitive to deltamethrin and other pyrethroids (Soderlund 2012). Over a range of relevant doses (0 to 100µM), we observed little cytotoxicity compared with vehicle controls following a 24 hr deltamethrin exposure (Fig. S1B and C). VGSC expression, specifically *Scn2a*, *Scn4a*, and *Scn9a* (but not *Scn3a*, *Scn5a*, *Scn8a*, *Scn10a*, or *Scn11a*), was greatly increased at higher doses at this timepoint (Fig. S1D), suggesting that the expression of these channels is indeed affected following deltamethrin exposure.

**Fig. 1.**
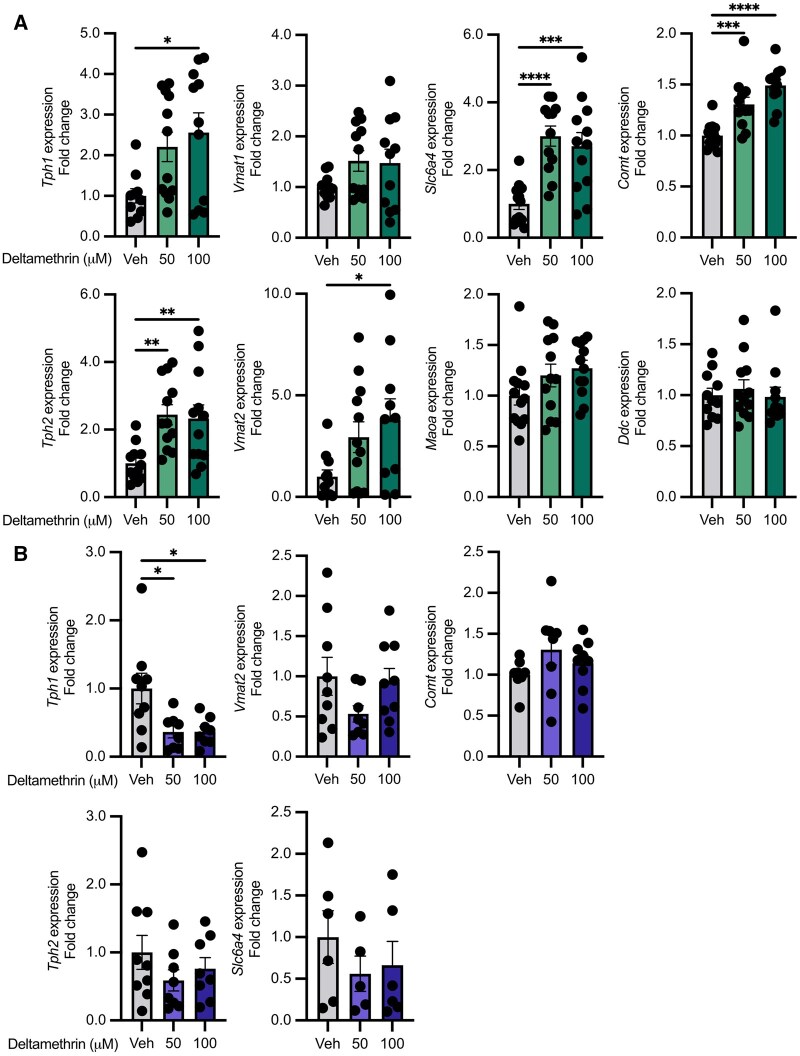
Deltamethrin dysregulates monoaminergic gene expression pathways in STC-1 cells in a calcium-dependent manner. A) Relative expression values expressed as fold change from STC-1 cells treated with DMSO vehicle alone (Veh), 50, or 100 µM deltamethrin for 24 h. B) Relative expression values expressed as fold change from STC-1 cells treated with DMSO vehicle alone (Veh), 50, or 100 µM deltamethrin for 24 h in calcium-free media. *n* = 9 to 12 (A) or *n* = 6 to 9 (B) independent culture wells per condition. Points represent independent wells (means of technical duplicates) and bars the mean ± SEM. Data compared by ordinary one-way ANOVA with Dunnett’s multiple comparisons test comparing each group to the control mean. *P ≤ 0.05, **P ≤ 0.005, ***P ≤ 0.001, ****P < 0.0001.

Because deltamethrin disrupts monoaminergic signaling in CNS neurons (Kirby et al. 1999; Elwan et al. 2006; Richardson et al. 2015; Mohammadi et al. 2019), we next sought to determine whether these pathways were similarly affected within these specific intestinal endocrine cells. Targeted gene expression analysis following 24 h of deltamethrin exposure revealed that deltamethrin significantly dysregulates components of monoamine function, in particular serotonin synthesis and release pathways that are central to EEC functions. This includes significant increases in *Tph1*, *Tph2*, *Vmat2*, *Slc6a4*, and *Comt* expression but no effects on *Vmat1*, *Maoa*, or *Ddc* (Fig. 1A). Exposure to a Type I pyrethroid, bioallethrin, did not induce cytotoxicity (Fig. S2A and B) but was similarly disruptive to monoaminergic gene expression levels (Fig. S2C), suggesting shared impacts to these pathways by representatives of both pyrethroid subtypes. Thus, pyrethroids transcriptionally dysregulate monoamine pathways in EECs.

VGSC activation, by pyrethroids and other agonists, leads to an influx of calcium to promote downstream signaling cascades (Soderlund et al. 2002). To determine whether the impacts we observed on gene expression in the monoamine pathways were dependent on calcium signaling, we performed identical deltamethrin exposures in the absence of extracellular calcium. Unlike deltamethrin exposures in calcium-replete media, in the absence of calcium, deltamethrin exposures did not result in the same widespread dysregulation of monoamine pathway genes (Fig. 1B). Excluding *Tph1*, expression of other genes, such as *Tph2*, *Vmat2*, *Slc6a4*, and *Comt* were unaffected. Therefore, the ability of deltamethrin to dysregulate these serotonergic and monoaminergic pathways in EECs is dependent on calcium signaling, and likely their canonical activation of VGSCs.

### Deltamethrin inhibits epinephrine-evoked GLP-1 release in STC-1 cells

**Fig. 2.**
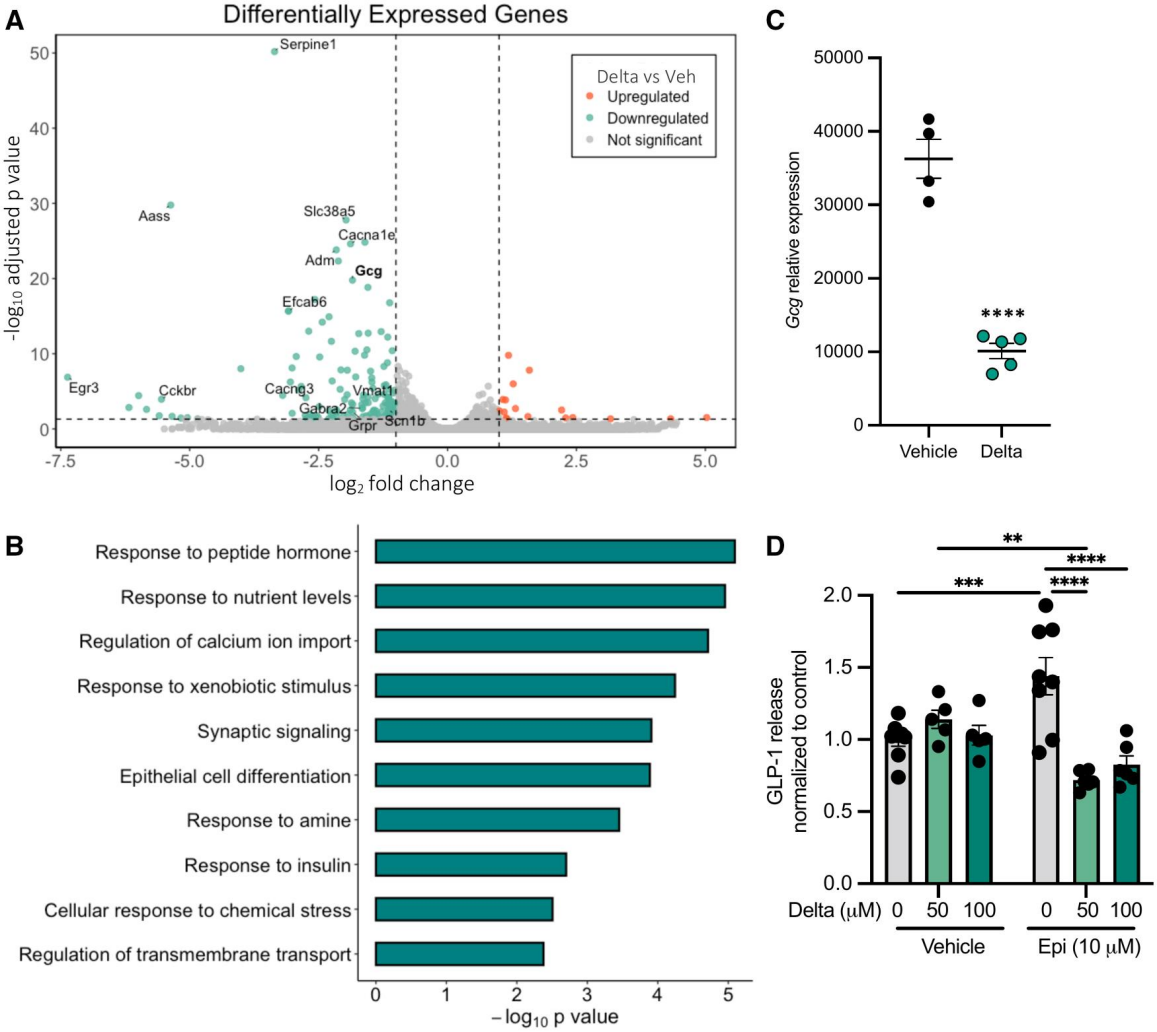
Deltamethrin downregulates gut hormonal expression pathways and inhibits epinephrine-induced GLP-1 release in STC-1 cells. A) Volcano plot of differentially expressed genes in STC-1 cells after 24 h of 100 µM deltamethrin exposure. Green dots represent significantly downregulated genes following deltamethrin exposure and red dots represent significantly upregulated genes following deltamethrin exposure. B) Significantly enriched pathways among downregulated DEGs according to Metascape. C) *Gcg* expression by bulk RNA sequencing in STC-1 cells after 24 h treatment with deltamethrin or vehicle control (adjusted *P*-value = 1.65e-20). D) GLP-1 release quantified by ELISA and normalized to control after 0, 50, or 100 µM deltamethrin treatment in the presence and absence of 10 µM epinephrine. A) Individual data points represent individual genes. B) Bars represent a set of differentially expressed genes associated with the corresponding enriched pathway. C and D) Data points represent averages of technical replicates from individual samples where (A through C) *n* = 4 to 5 and (D) *n* = 5 to 8 and depicted as mean ± SEM. (A through C) Differential genes determined by corrected *P*-value of 0.005 and an absolute log2 fold change threshold of 1 and (B) selections for enrichments with a stringent *P*-value threshold of 0.02. (D) Data compared by ordinary two-way ANOVA with Tukey’s multiple comparisons test comparing means all means to one another. **P < 0.005, ***P < 0.001, ****P < 0.0001.

Other than monoamines, EECs produce a variety of neuropeptide hormones (McCarthy et al. 2015), which may also be affected by pyrethroid exposure. To investigate the impact of deltamethrin on STC-1 transcriptional state, particularly on the expression of hormonal pathways, we performed bulk RNA sequencing on STC-1 cells treated with vehicle or 100 µM deltamethrin for 24 h. We observed 159 differentially expressed genes (DEGs), 142 of which were significantly downregulated in response to deltamethrin treatment (Fig. 2A and our Supplemental Datasets [Zenodo #14803680 and #1477919]). Pathway analysis revealed that “Response to peptide hormone,” was one of the most significantly enriched pathways among the downregulated DEGs, along with “Response to nutrient levels” (Fig. 2B and Zenodo #14803680), suggesting that deltamethrin indeed transcriptionally dysregulates genes involved in hormonal activities. Notably, the gene encoding preproglucagon (*Gcg*), the precursor to the EEC-derived gut hormone GLP-1, is significantly downregulated with deltamethrin treatment (Fig. 2C). Given that STC-1 cells robustly produce GLP-1 in response to various stimuli (Reimann et al. 2006), we then assessed how deltamethrin functionally impacts EEC neuropeptide signaling by measuring GLP-1 release. STC-1 cells were treated with epinephrine or glucose, 2 compounds known to stimulate GLP-1 release in these cells (Harada et al. 2015; McCarthy et al. 2015), resulting in a dose-dependent increase in GLP-1 release, as anticipated (Fig. S3A and B). To determine how deltamethrin may affect the ability of EECs to respond to these stimuli, cells were pre-treated with deltamethrin prior to an epinephrine stimulus. Although deltamethrin alone did not affect GLP-1 release (Fig. 2D), pre-treatment with deltamethrin significantly inhibited epinephrine-mediated GLP-1 release (Fig. 2D). These data indicate that deltamethrin interferes with the ability of EECs to respond to key modulatory signals, resulting in significant functional dysregulation across monoamine and GLP-1 signaling pathways.

### Oral deltamethrin exposure induces acute constipation in mice

**Fig. 3.**
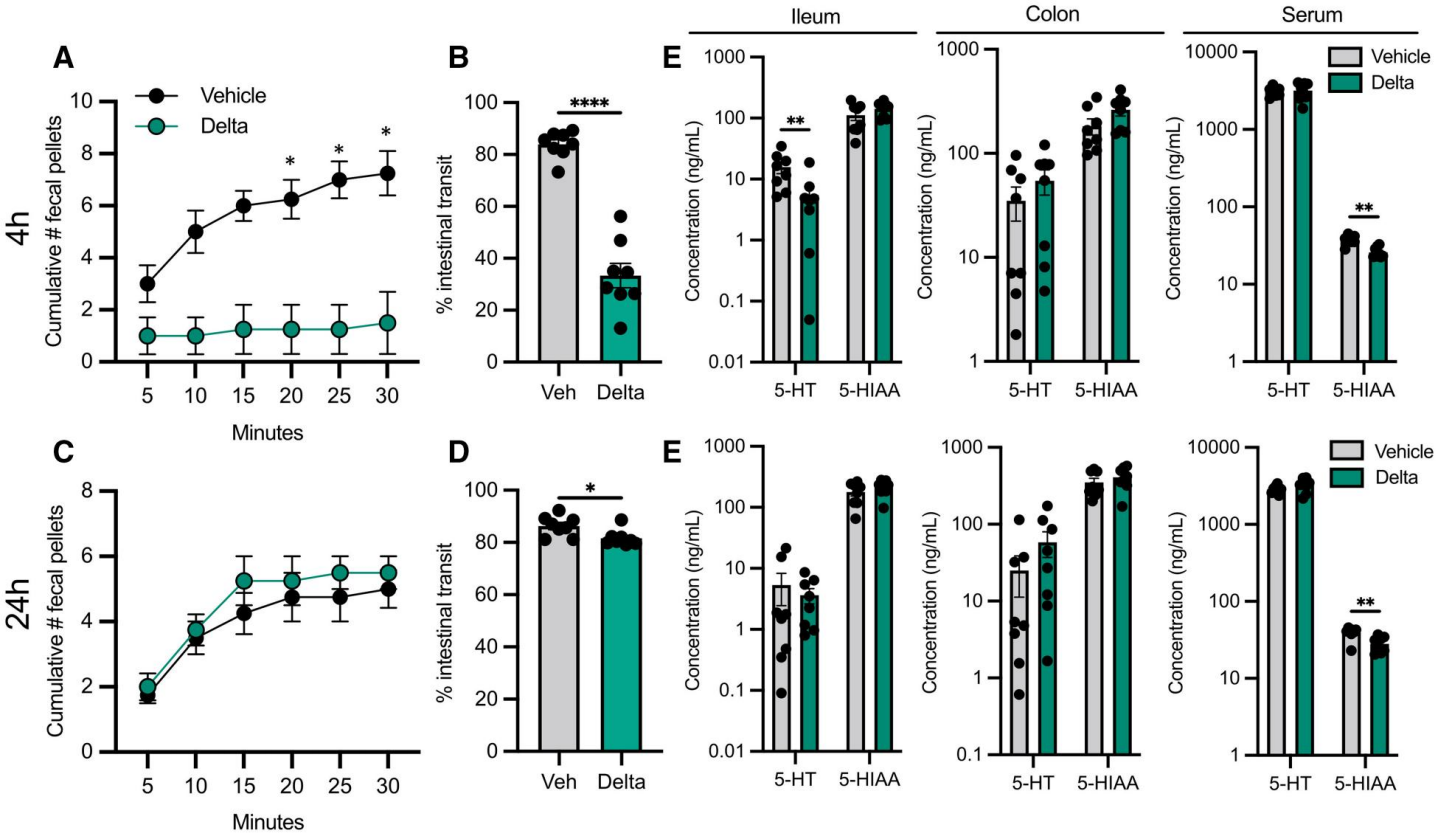
Oral deltamethrin exposure induces acute intestinal dysmotility and limits serotonin production. A) Fecal output (# pellets produced in 30 min) after 4 h deltamethrin or vehicle exposure. B) The % intestinal transit of red carmine dye in the GI tract of 4 h vehicle or deltamethrin-treated mice immediately after sacrifice. C) Fecal output after 24 h deltamethrin or vehicle exposure. D) The % intestinal transit of 24 h vehicle or deltamethrin-treated mice. E and F) Peripheral serotonin (5-HT) and 5-HIAA levels at 4 h (E) or 24 h (F) post vehicle or deltamethrin exposure in ileum, colon, and serum, as measured by HPLC. All data points represent averages of biological (A and C) or technical (B and D through F) replicates where *n* = 4 (A and C) or *n* = 8 (B and D through F) per group. Data are depicted as mean ± SEM and compared by two-way repeated measures ANOVA with Šídák’s multiple comparisons test between treatment groups (A and C), Mann–Whitney test for non-normally distributed data (D through F), or two-tailed unpaired student’s t-tests (B, E, and F). *P < 0.05, **P < 0.005, ****P < 0.0001.

Given the significant impact on motility-regulating monoamine and GLP-1 pathways we observed following deltamethrin treatment of EEC-like cells in culture, we sought to determine potential intestinal functional impacts in vivo. We performed a battery of GI assessments in ad-lib fed mice following an oral exposure to low-dose deltamethrin (3 mg/kg body weight). After an acute exposure, 4 h post oral gavage (serum C_max_ time), we observed that deltamethrin-treated male mice displayed significantly reduced fecal pellet output compared with those treated with corn oil vehicle (Fig. 3A). This acute effect of oral deltamethrin exposure was not observed in female mice (Fig. S4). Further evaluation of male mice, using a terminal dye transit assay confirmed that total intestinal motility was significantly delayed after deltamethrin exposure (Fig. 3B). By 24 h post-exposure, fecal output in treated male animals was restored, and intestinal transit was improved, albeit still impaired compared with vehicle controls, suggesting that deltamethrin’s effects, whereas drastic acutely in male mice, are transient (Fig. 3C and D). These data clearly demonstrate that a single, low-dose, orally administered deltamethrin exposure is sufficient to induce a constipation-like phenotype in mice.

Because we observed a striking in vivo intestinal motility phenotype that correlated with dysregulation of monoamine pathways in EECs in culture, we next measured circulating and tissue-resident serotonin levels. In deltamethrin-treated male mice, we detected significantly lower concentrations of serotonin (5-HT) in the ileum (∼65% decrease post-treatment) and lower 5-hydroxyindoleacetic acid (5-HIAA, the major serotonin metabolite) levels in serum (∼31% decrease), with no difference in colonic concentrations at acute (4 h) timepoints post-exposure (Fig. 3E). At 24 h post deltamethrin exposure, we observed only a significant reduction in serum 5-HIAA (∼28% reduction), with no differences between treatment groups in the ileum or colon (Fig. 3F). These data together demonstrate that oral deltamethrin exposure significantly and acutely disrupts intestinal motility and accompanying peripheral monoamine signaling pathways.

### Deltamethrin interferes with nutrient-stimulated intestinal hormone signaling in vivo

**Fig. 4.**
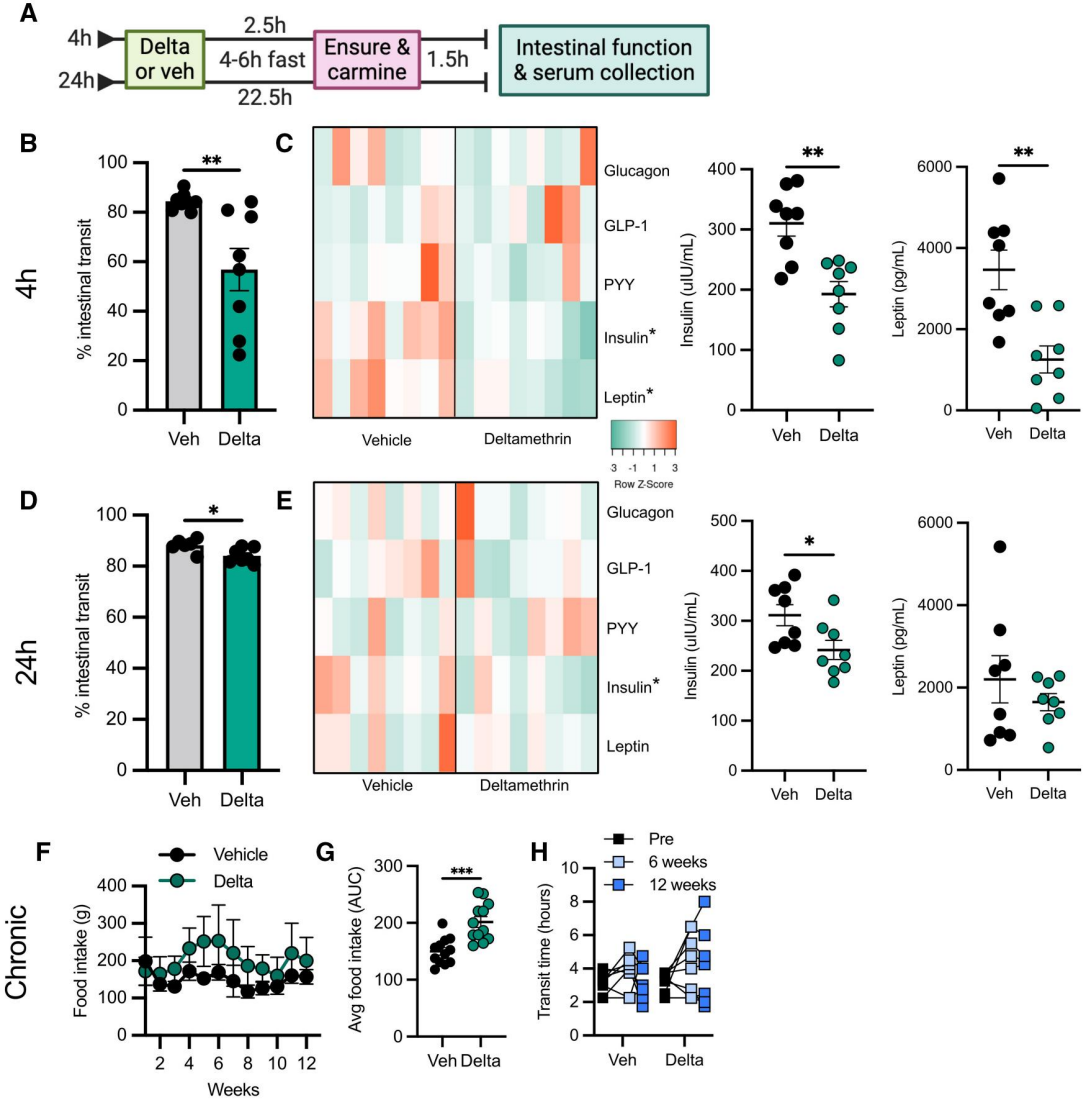
Deltamethrin inhibits release of intestinal hormones following a mixed-meal nutrient-stimulus. A) Schematic representation of the experimental timeline. B) % intestinal transit of carmine red dye 4 h after exposure to either vehicle or deltamethrin and measured upon sacrifice. C) Accompanying gut hormone heatmap in serum after 4 h exposure to vehicle or deltamethrin, determined by multiplexed ELISA. Insulin and leptin are depicted separately. D) % intestinal transit of carmine dye after 24 h exposure to vehicle or deltamethrin. E) Corresponding quantification of gut hormone levels in serum by heatmap after 24 h exposure to vehicle or deltamethrin, with insulin and leptin depicted separately. F) Average weekly food intake after chronic exposure to vehicle (black) or deltamethrin (green). G) Average food intake expressed as area under the curve for vehicle- and deltamethrin-treated mice. H) Total intestinal transit time in vehicle or deltamethrin-treated mice after 0 (black), 6 (light blue), or 12 weeks (blue) of exposure. C and E) Concentration units are in pM for GLP-1 (active) and glucagon, uIU/ml for insulin, and pg/ml for leptin and PYY (total). B through H) All data points represent individual mice (B, D, and H), their averages after technical duplicates (C and E), or the average of 2 cages of 4 mice per cage (F and G), where *n* = 8 per group. Bars are depicted as mean ± SEM and compared by unpaired student’s *t*-test (B, C, E, and G), Mann–Whitney nonparametric tests for non-normally distributed data (glucagon and PYY in C and D), or a two-way repeated measures ANOVA (F and H). *P < 0.05, **P < 0.005, ***P < 0.0005.

GLP-1 is released in vivo in response to glucose and other nutritional stimuli (Reimann et al. 2006). Given our observations that deltamethrin inhibits GLP-1 release in EECs in vitro, we assessed how deltamethrin modulates intestinal hormones, including GLP-1, in response to a high-nutrient, mixed-meal stimulus in vivo. Male mice treated with either deltamethrin or a corn oil vehicle were fasted for 4 to 6 h immediately prior to stimulation with a mixed-meal nutrient oral bolus (Fig. 4A). Similar to our observations in fed mice (Fig. 3B and D), oral deltamethrin exposure resulted in an acute but transient intestinal dysmotility compared with vehicle controls, even in the presence of a mixed meal (Fig. 4B and D). To determine the extent to which deltamethrin may interfere with GI hormone signaling in vivo, we measured an array of circulating intestinal neuropeptide hormones. Although GLP-1 concentrations were not affected in vivo, we observed significantly less circulating insulin and leptin following acute deltamethrin exposure (Fig. 4C). This effect was transient, with concentrations similar to vehicle controls 24 h post-exposure (Fig. 4E). Because both insulin and leptin contribute to satiety, and deltamethrin inhibited their release following a mixed meal, we tested whether deltamethrin modified food intake. Using a chronic treatment paradigm that allows the capture of weekly food intake, we observe an increase in food intake in deltamethrin-exposed mice (Fig. 4F and G), as well as qualitatively impaired GI transit (Fig. 4H). As a whole, these data emphasize a role for deltamethrin in perturbing broad GI functions through neuropeptide modulation.

## Discussion

EECs are among the first cell types to interact with ingested environmental toxicants. Their anatomical location at the interface between the intestinal luminal environment and the nervous system, in conjunction with their neuron-like properties, underscores their vulnerability to neurotoxic compounds. Because pyrethroids are highly prevalent and a common route of their exposure is ingestion (ATSDR 2003), they are a relevant exposure to impact EEC function and intestinal physiology. Here, we show that the pyrethroid deltamethrin affects the expression of monoaminergic pathways in EECs in vitro and induces in vivo perturbations to the GI system that are associated with dysregulation of EEC functions, including serotonergic signaling and neuropeptide hormone release. These findings not only establish EECs as vulnerable to pyrethroid toxicity but also provide insight into the mechanisms by which deltamethrin alters intestinal physiology, which may underlie known GI dysfunctions of exposure-associated diseases.

Several studies report various GI disturbances following high-dose or chronic pyrethroid exposures in both animals and humans (He et al. 1989; ATSDR 2003; Chrustek et al. 2018; Ma et al. 2023; Scheepers et al. 2023). These include nausea, intestinal pain, dysmotility, and GI inflammation—pathologies that overlap with clinical features of irritable bowel syndrome, which is associated with pyrethroid exposure (Rueda-Ruzafa et al. 2023), and IBD (Ma et al. 2023). As dysregulation of both serotonin and neuropeptide signaling contribute to these diseases and pathologies (Sikander et al. 2009; Zietek and Rath 2016; Ahmed and Ahmed 2019; Kwon et al. 2019; Grondin and Khan 2024), our data support a direct pathological contribution of deltamethrin exposure to the EEC dysfunctions that characterize these intestinal diseases.

We observe pyrethroid-induced suppression of physiologically stimulated GI hormones GLP-1, insulin, and leptin, which each have broad actions in GI physiology and metabolism (Yarandi et al. 2011; Campbell and Drucker 2013; Ussar et al. 2017). Pyrethroid exposure is linked to impairments in glucose metabolism, including diabetes (Wang et al. 2011; Hansen et al. 2014; Furlong et al. 2020; Jia et al. 2022), with mixed findings to obesity (Tsakiridis et al. 2021; Zuo et al. 2022). Interestingly, serotonin has also been implicated as a contributor to obesity and diabetes (Yabut et al. 2019). Given our observations that deltamethrin directly impacts both neuropeptide hormones and serotonin pathways in EECs, it suggests that EEC dysfunctions may be a link between pyrethroid toxicity and further metabolic disruption in the GI tract and systemically.

More broadly, pyrethroid exposure is linked with varying degrees of strength to neurodevelopmental disorders, such as autism and attention-deficit hyperactivity disorder (Richardson et al. 2015; Vester et al. 2019; Curtis et al. 2023) and neurodegenerative outcomes relevant to both PD (Kirby et al. 1999; Karen et al. 2001; Elwan et al. 2006; Xiong et al. 2016) and amyotrophic lateral sclerosis (Doi et al. 2006), many of which involve established disruptions to monoaminergic pathways, including serotonin (Muller et al. 2016; Vermeiren et al. 2018). PD in particular presents with significant GI dysfunctions (Liddle 2018), though the specific intestinal pathologies that underlie these are not well described. Constipation itself is a prevalent prodrome of PD (Klingelhoefer and Reichmann 2015; Savica et al. 2018; O'Day et al. 2022; Camerucci et al. 2023), and pyrethroids are epidemiologically linked to PD risk (Furlong et al. 2015; Ritz et al. 2016; Paul et al. 2023). It is therefore tempting to speculate that environmental exposures, such as to pyrethroids, trigger early pathologies at the site of exposure within the GI tract. In addition, emerging experimental and clinical data implicate GLP-1 signaling in limiting PD pathologies (Harkavyi et al. 2008; Li et al. 2009; Aksoy et al. 2017; Athauda et al. 2017; Meissner et al. 2024). Clinical studies have recently demonstrated benefit of systemic GLP-1 agonists for PD symptoms (Athauda et al. 2017; Meissner et al. 2024). Although the protective mechanisms are currently unclear (Verma and Goyal 2024), there are known roles for GLP-1 and insulin signaling in PD and other neurological disease risk (Han et al. 2023; Ruiz-Pozo et al. 2023; Zheng et al. 2024).

Our data demonstrate that deltamethrin interferes with GLP-1 release and dampens a nutrient-evoked insulin response, which may contribute to the association between pyrethroid exposure and these diseases. Serotonergic signaling in the CNS is also impacted post-pyrethroid exposure (Hossain et al. 2013; Rodríguez et al. 2016; Maximiliano et al. 2024), further validating our observations that these insecticides may contribute to GI dysfunctions via similar mechanisms. Our data herein support the notion that pyrethroid exposures are sufficient to induce dysfunction within vulnerable intestinal cells that lead to relevant prodromal features of these and other diseases.

### Limitations of this study

Both human and murine EECs display significant similarity in their transcriptional repertoires, including the expression of pyrethroid-sensitive VGSCs (Roberts et al. 2019; Stanford et al. 2020). However, we appreciate that studies in murine models may not fully replicate human intestinal responses to these exposures. In addition, we focused our studies on male mice, based on our initial assessment that female mice did not display an acute constipation-like phenotype post-deltamethrin exposure (Fig. S4). Our data provide a foundation for future studies to directly compare physiological outcomes to these exposures based on sex. Lastly, we chose to orally administer deltamethrin in a corn oil vehicle, as a relevant food-grade matrix. Alternative vehicles (e.g. DMSO, ethanol, methylcellulose, glycerol formal) or routes of exposure (e.g. inhalational, systemic) may differentially impact toxicokinetic properties and the intestinal outcomes we observe (Crofton et al. 1995; Mortuza et al. 2018). Nonetheless, our data herein provide a foundational dataset on EEC functional and transcriptional responses, as well as acute intestinal physiologies in response to a low-dose, oral pesticide exposure.

## Conclusion

This study directly addresses pyrethroid-monoamine interactions within the GI tract, a critical step toward understanding the broad health impacts of pyrethroid exposure on intestinal physiology that is linked to many pyrethroid-associated diseases. We provide compelling evidence that the pyrethroid deltamethrin alters intestinal pathways important for serotonin trafficking in EECs, acutely impairs intestinal motility, and diminishes intestinal hormone production in response to physiological stimuli. Our data highlight the continued need to study toxicological impacts in the GI tract that may be associated with long-term exposure to these prevalent chemicals and underlie those diseases epidemiologically linked to exposure.

## Supplementary Material

kfaf076_Supplementary_Data

## Data Availability

All numerical data, analyzed RNAseq files, and statistical outputs are available in either the Supplemental Source Data File associated with this manuscript or the Zenodo repository under accession numbers 14775919 and 14803680 (doi.org/10.5281/zenodo.14775919 and doi.org/10.5281/zenodo.14803680), as indicated. Raw RNAseq read files are available at NIH SRA database accession PRJNA1218260.
