# Supplementary material for: The pyrethroid insecticide deltamethrin disrupts neuropeptide and monoamine signaling pathways in the gastrointestinal tract
Source: Toxicol Sci. 2025 Jun 26;207(1):208–20. doi: 10.1093/toxsci/kfaf076 (PMC12448213; doi:10.1093/toxsci/kfaf076)
Supplement: kfaf076_Supplementary_Data [file kfaf076_supplementary_data.pdf]

The pyrethroid insecticide deltamethrin disrupts neuropeptide and monoamine signaling pathways in the gastrointestinal tract

Alexandria C. White[1,2], Ian N. Krout[1,2], Sabra Mouhi[1], Lisa Blackmer-Raynolds[1,2], Jianjun Chang[1], Sean D. Kelly[1], W. Michael Caudle[3], Timothy R. Sampson*[1,2]

[1]Dept of Cell Biology; Emory University School of Medicine; Atlanta GA USA 30329

[2]Aligning Science Across Parkinson's (ASAP) Collaborative Research Network; Chevy Chase MD 20815

[3]Gangarosa Dept of Environmental Health, Rollins School of Public Health; Emory University; Atlanta GA 30329

*To whom correspondence should be addressed: trsamps@emory.edu

Supplementary Information

Supplementary Figure S1
Supplementary Figure S2
Supplementary Figure S3
Supplementary Figure S4
Supplementary Table S1

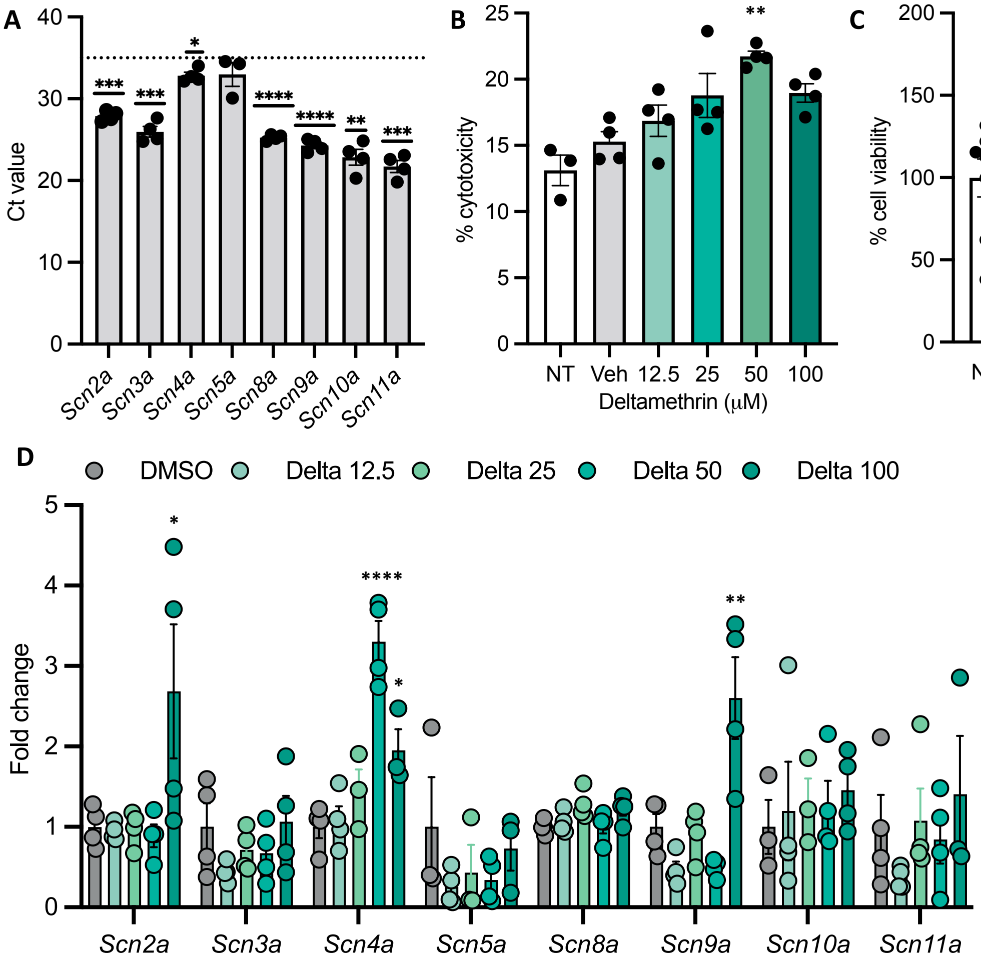

Supplementary Fig. S1. STC-1 cells express deltamethrin-sensitive voltage-gated sodium channels. A Ct values of each voltage-gated sodium channel (VGSC) subtype expressed in STC-1 cells. **B** Quantification of % cytotoxicity by lactate dehydrogenase (LDH) detection assay from STC-1 cells treated with 0, 12.5, 25, 50, or 100 μM deltamethrin for 24h. **C** Quantification of % cell viability by MTT assay from STC-1 cells treated with 0, 12.5, 25, 50, or 100 μM deltamethrin for 24h. **D** Fold change of different VGSC subtypes from STC-1 cells treated with 0, 12.5, 25, 50, or 100 μM deltamethrin for 24h, as determined by qPCR. **A-D** All data points represent averages of technical duplicates from an individual well where n = 3-4 (**A, B, D**) or n = 8 (**C**). Data are depicted as mean $\pm$ SEM and compared by ordinary one-way ANOVA with Dunnett's multiple comparisons tests (**B-D**) or one-sample t-test against a Ct cutoff value of 35 (**A**), represented by a dashed line. *p<0.05, **p< 0.005, ****p<0.0001.

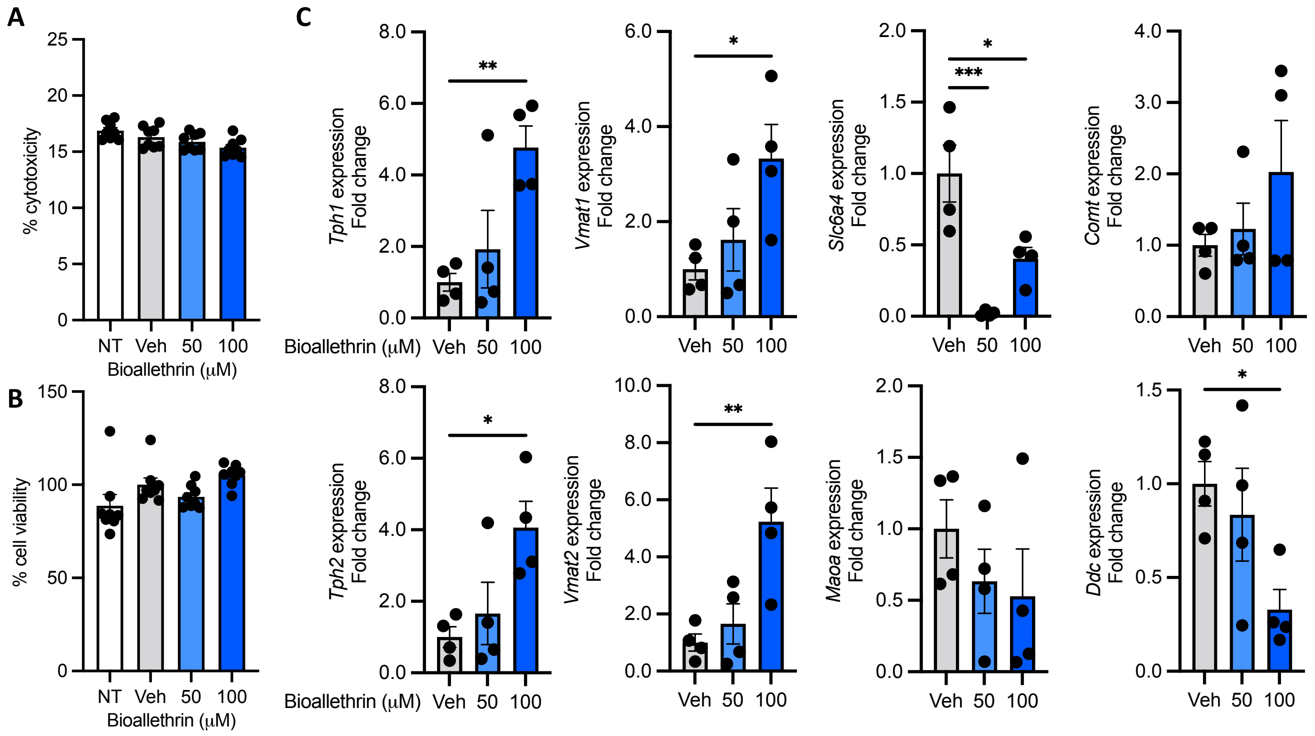

Supplementary Fig. S2. The pyrethroid bioallethrin dose-dependently disrupts transcriptional serotonergic pathways. A Quantification of % cytotoxicity by lactate dehydrogenase (LDH) detection assay from STC-1 cells treated with 0, 50, or 100 μM bioallethrin for 24h. **B** Quantification of % cell viability by MTT assay from STC-1 cells treated with 0, 50, or 100 μM bioallethrin for 24h. **C** Relative expression values expressed as fold change from STC-1 cells treated with 0, 50, or 100 μM bioallethrin for 24h. **A-C** All data points represent averages of technical duplicates from an individual well where n = 8 (**A, B**) or n = 4 (**C**). Data are depicted as mean ± SEM and compared by ordinary one-way ANOVA with Dunnett's multiple comparisons tests (**A-C**). *$p<0.05$, **$p< 0.005$, ***$p<0.001$.

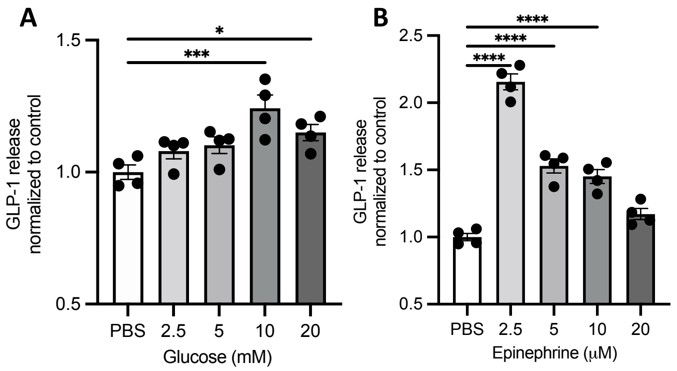

Supplementary Fig. S3. Glucose and epinephrine evoke GLP-1 release in STC-1 cells. A Quantification of GLP-1 release by ELISA from STC-1 cells treated with 0, 2.5, 5, 10, or 20 mM glucose for 1h. **B** Quantification of GLP-1 release by ELISA from STC-1 cells after treatment with 0, 2.5, 5, 10, or 20 μM epinephrine for 1h. **A-B** All data points represent averages of technical duplicates from individual samples where n = 3-4 per group. Data are depicted as mean $\pm$ SEM and compared by ordinary one-way ANOVA with Dunnett's multiple comparisons test comparing each group to the control (**A-B**). *p<0.05, ***p<0.001, ****p<0.0001.

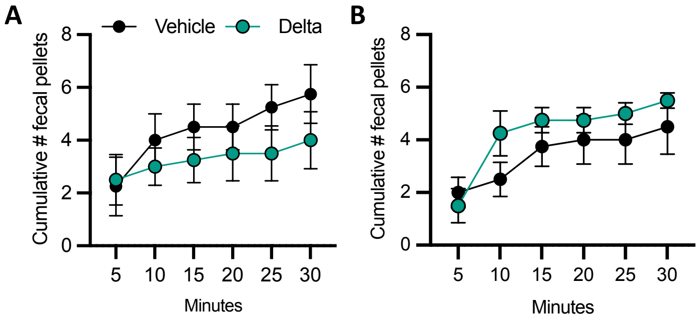

Supplementary Fig. S4. Deltamethrin does not induce intestinal dysmotility in female mice. A Fecal output (cumulative # fecal pellets produced in 30 mins) after 4h of deltamethrin exposure. **B** Fecal output after 24h deltamethrin exposure. **A-B** All data points represent averages of biological replicates from individual samples where n = 4 per group. Data are depicted as mean ± SEM and compared by Two-way repeated measures ANOVA with Šídák's multiple comparisons test between treatment groups at each time point (**A-B**).

Supplementary Table 1. Key resource table

RESOURCE TYPE	RESOURCE NAME	SOURCE	IDENTIFIER	NEW/ REUSE
Dataset	Bulk RNA sequencing data	NIH SRA	PRJNA1218260	new
Dataset	Bulk RNA sequencing methods & analysis	Zenodo	10.5281/zenodo.14775918	new
Dataset	Data & statistical outputs	Zenodo	10.5281/zenodo.14803680	new
Software/code	Prism v10.4.1	GraphPad	https://www.graphpad.com/	reuse
Software/code	R v3.5.0	The Comprehensive R Archive Network	https://cran.r-project.org/	reuse
Software/code	R Studio v4.4.2 - "Pile of Leaves"	Posit	https://posit.co/download/rstudio-desktop/	reuse
Software/code	7900 SDS 2.4	Applied Biosystems	https://www.thermofisher.com/us/en/home/technical-resources/software-downloads/applied-biosystems-7900ht-fast-real-timespcr-system.html	reuse
Software/code	Heatmapper	PMID: 27190236	http://heatmapper.ca/	reuse
Protocol	STC-1 cell culture	protocols.io	dx.doi.org/10.17504/protocols.io.j8nlk98kwv5r/v1	reuse
Protocol	Dye-based intestinal motility	protocols.io	dx.doi.org/10.17504/protocols.io.eq2ly6pjwgx9/v1	new
Protocol	Total GI transit assay	protocols.io	dx.doi.org/10.17504/protocols.io.14egn9676l5d/v1	reuse
Protocol	Fecal Output	protocols.io	dx.doi.org/10.17504/protocols.io.rm7vzj3j5lx1/v1	reuse
Protocol	LDH cytotoxicity assay	protocols.io	dx.doi.org/10.17504/protocols.io.261ger51yl47/v1	reuse
Protocol	MSD Multiplexed ELISA	protocols.io	dx.doi.org/10.17504/protocols.io.5jyl8dq5rg2w/v1	reuse
Protocol	Oral exposures	protocols.io	dx.doi.org/10.17504/protocols.io.3byl4w198vo5/v1	reuse
Protocol	HPLC monoamine detection assay	protocols.io	dx.doi.org/10.17504/protocols.io.q26g7m9m1gwz/v1	reuse
Protocol	MTT cell viability assay	protocols.io	dx.doi.org/10.17504/protocols.io.5jyl8dky8g2w/v1	reuse
Protocol	GLP-1 ELISA assay	protocols.io	dx.doi.org/10.17504/protocols.io.j8nlkokm6v5r/v1	reuse
Protocol	RNA extraction	protocols.io	dx.doi.org/10.17504/protocols.io.e6nvwdyn7lmk/v1	reuse
Protocol	cDNA synthesis	protocols.io	dx.doi.org/10.17504/protocols.io.14egn9676l5d/v1	reuse
Protocol	RT qPCR	protocols.io	dx.doi.org/10.17504/protocols.io.36wgqdn1ovk5/v1	new

Experimental model: Cell line	STC-1 cell line	ATCC	Cat #CRL-3254; RRID:CVCL_J405	reuse
Experimental model: Organism/strain	C57BL/6J mice	Jax	RRID:IMSR_JAX:000664	reuse
Chemical, peptide, or recombinant protein	Deltamethrin	Chem Service	Cat #N-11579-250MG	reuse
Chemical, peptide, or recombinant protein	Bioallethrin	AccuStandard	Cat # P-664N	reuse
Chemical, peptide, or recombinant protein	Glucose	Sigma	Cat #G8270-1KG	reuse
Chemical, peptide, or recombinant protein	Epinephrine	Sigma	Cat #E4250-1G	reuse
Chemical, peptide, or recombinant protein	Carmine red dye	Sigma	Cat #C1022	reuse
Chemical, peptide, or recombinant protein	Ensure Original mixed-meal nutrient drink	Abbott Pharmaceuticals	SKU #: 57243	reuse
Chemical, peptide, or recombinant protein	Corn oil	Mazola	UPC code: 7 61720 98749 0	reuse
Critical commercial assay	GLP-1 ELISA kit	Millipore	Cat #EGLP-35K	reuse
Critical commercial assay	LDH cytotoxicity kit	ProMega	Cat # G1780	reuse
Critical commercial assay	U-PLEX Metabolic Hormones Combo 1 for mouse	Meso Scale Discovery	Cat # K15306K-2	reuse
Critical commercial assay	MTT cell viability kit	Invitrogen	Cat #V13154	reuse
Oligonucleotide	Tph1 qPCR primer forward	IDT	CCATCTTCCGAGAGCTAAACAAA	new
Oligonucleotide	Tph1 qPCR primer reverse	IDT	TCTTCCCGATAGCCACAGTATT	new
Oligonucleotide	Tph2 qPCR primer forward	IDT	TCGAAATCTTCGTGGACTGCG	new
Oligonucleotide	Tph2 qPCR primer reverse	IDT	CGGATTCAGGGTCACAATGGT	new
Oligonucleotide	Vmat1 qPCR primer forward	IDT	GTCCCGGAAGCTGGTGTTG	new
Oligonucleotide	Vmat1 qPCR primer reverse	IDT	ACAGTGAGCAGCATATTGTCC	new
Oligonucleotide	Vmat2 qPCR primer forward	IDT	CGCAAGCTGATCCTGTTCATC	new
Oligonucleotide	Vmat2 qPCR primer reverse	IDT	ACGACGGTGAGCAGCATGT	new
Oligonucleotide	Slc6a4 qPCR primer forward	IDT	TATCCAATGGGTACTCCGCAG	new
Oligonucleotide	Slc6a4 qPCR primer reverse	IDT	CCGTTCCCCTTGGTGAATCT	new
Oligonucleotide	Ddc qPCR primer forward	IDT	TAGCTGACTATCTGGATGGCAT	new
Oligonucleotide	Ddc qPCR primer reverse	IDT	GTCCTCGTATGTTTCTGGCTC	new

Oligonucleotide	Maoa qPCR primer forward	IDT	GTGAATGTCAATGAGCG TCTAGT	new
Oligonucleotide	Maoa qPCR primer reverse	IDT	TCAACAGGGATCTCTTT TCCCA	new
Oligonucleotide	Comt qPCR primer forward	IDT	CTGGGGGTTGGTGGCT ATTG	new
Oligonucleotide	Comt qPCR primer reverse	IDT	CCCACTCCTTCTCTGAG CAG	new
Oligonucleotide	Gapdh qPCR primer forward	IDT	TGGCCTTCCGTGTTCCT A	new
Oligonucleotide	Gapdh qPCR primer reverse	IDT	GAGTTGCTGTTGAAGTC GCA	new
